# Efficacy of a hybrid case-based learning and simulated clinical encounter model versus lecture-based learning in dental education

**DOI:** 10.1186/s12909-026-08937-x

**Published:** 2026-03-09

**Authors:** Lijia Rao, Jiahua Wu, Bixia Deng, Yubing Hong, Juan Wu, Yiming Chen, Mu Chen

**Affiliations:** Department of Stomatology, Affiliated Nanshan Hospital of Shenzhen University, No.89 Taoyuan Road, Nanshan District, Shenzhen, Guangdong Province 518052 P.R. China

**Keywords:** Dental Education, Case-Based Learning (CBL), Scene Simulation Teaching (SST), Dental simulator, ICAP Framework, Miller’s Pyramid, Clinical Competence

## Abstract

**Background:**

Current dental education faces challenges in bridging the gap between theoretical knowledge and clinical application. Traditional Lecture-Based Learning (LBL) often fails to alleviate student anxiety or foster deep cognitive engagement. This study explores the efficacy of a hybrid teaching model that integrates Case-Based Learning (CBL) and Scene Simulation Teaching (SST) with dental simulator practice, comparing it with the traditional Lecture-Based Learning (LBL) model.

**Methods:**

A randomized controlled study was conducted with 20 fifth-year undergraduate dental students from the 2018 cohort at the Affiliated Nanshan Hospital of Shenzhen University. Participants were randomly allocated to either the experimental group (Hybrid CBL-SST-Simulator) or the control group (Traditional LBL). The Endodontics course served as the context. Outcomes were assessed across four dimensions: theoretical knowledge (written exam), clinical thinking (case analysis), doctor-patient communication (SEGUE Framework), and clinical skills (preparation of Class II cavity and access opening).

**Results:**

Students in the Hybrid group demonstrated significantly superior performance in clinical thinking (87.40 ± 5.20 vs. 73.90 ± 8.41, *P* < 0.05) and doctor-patient communication skills (17.80 ± 1.89 vs. 14.20 ± 2.18, *P* < 0.05) compared to the LBL group. However, there were no statistically significant differences regarding theoretical knowledge scores (*P* > 0.05) or clinical operational skills (*P* > 0.05).

**Conclusions:**

The hybrid CBL-SST model significantly enhances clinical reasoning and communication skills by promoting “Interactive” and “Constructive” learning behaviors. While it does not surpass traditional methods in rote knowledge or pure manual dexterity, it effectively bridges the gap between theory and practice. This approach offers a promising strategy for preclinical dental education.

**Supplementary Information:**

The online version contains supplementary material available at 10.1186/s12909-026-08937-x.

## Background

Dentistry is a discipline that demands a seamless integration of theoretical knowledge, manual dexterity, and interpersonal skills. However, a persistent challenge in dental education is the theory-practice gap. Recent studies have indicated that dental students often experience significant anxiety and lack of confidence in diagnosis and treatment planning when transitioning from preclinical to clinical phases [1, 2]. Traditional Lecture-Based Learning (LBL), while effective for information dissemination, often places students in a passive role, leading to fragmented knowledge retention and difficulty in applying concepts to real-world scenarios.

To address these limitations, educators have turned to active learning methodologies. Case-Based Learning (CBL) contextualizes knowledge through patient narratives [3], while Scene Simulation Teaching (SST) allows students to practice professional roles in realistic, safe environments [4]. Recent research underscores the growing efficacy of these approaches. For instance, Wang et al. demonstrated that CBL significantly enhances diagnostic accuracy in fluorosis grading [5], while Khattak et al. reported boosted confidence in operative procedures such as cavity preparation [6]. Similarly, SST has proven superior in training emergency management skills [7] and refining complex treatment planning [8]. Integrating CBL and SST with high-fidelity dental simulators, these methods form a comprehensive “Simulated Clinical Encounter.” While systematic reviews confirm a global shift toward these immersive strategies to improve learner confidence and procedural safety, understanding the theoretical basis for integrating these methods is essential.

Theoretically, this integration aligns with the Interactive–Constructive–Active–Passive (ICAP) framework, which posits that shifting students from Passive listening to Active, Constructive, and Interactive modes leads to deeper learning outcomes [9]. Furthermore, Miller’s Pyramid of Clinical Competence suggests that education must move beyond “Knows” (knowledge) and “Knows How” (competence) to “Shows How” (performance) [10].

While individual components have been studied, the synergistic effect of integrating CBL, SST, and Simulator practice into a single hybrid model compared to traditional LBL remains under-explored. This study aims to evaluate the efficacy of this hybrid model in an undergraduate Endodontics course, hypothesizing that it will improve clinical thinking and communication skills without compromising theoretical knowledge or manual skills.

## Methods

### Study Design and Participants

This randomized controlled pilot study was conducted in accordance with the Declaration of Helsinki and was approved by the Ethics Committee of Affiliated Nanshan Hospital of Shenzhen University (Approval No. 2023-EDU-015). A total of 20 fifth-year undergraduate dental students from the 2018 cohort were recruited.

The inclusion criteria were: (1) completion of all theoretical courses prior to clinical practice; (2) attendance in the same foundational courses to ensure consistent baseline knowledge; and (3) passing the undergraduate theoretical examination with Endodontics scores > 70/100.

The exclusion criteria were: (1) disciplinary violations resulting in suspension; and (2) withdrawal from the training due to personal reasons.

Participants were randomly allocated to either the experimental group (Group A, *n* = 10) or the control group (Group B, *n* = 10) using stratified randomization based on gender and age to ensure baseline equivalence.

### Intervention

The study focused on two modules of *Endodontics*: “Caries and Pulp Disease”. Group A received the hybrid CBL-SST-Simulator teaching, while Group B received traditional LBL teaching. The training program consisted of weekly sessions over a period of 6 weeks. Each session had a duration of 2 h. Assessments were administered one week after course completion to evaluate learning outcomes.

#### Control group (traditional LBL Teaching)

Group B received traditional Lecture-Based Learning (LBL) [11]. The teaching process followed a standard sequence:

1. Theoretical Instruction: Instructors utilized multimedia courseware (PowerPoint and videos) to explain theoretical knowledge according to the syllabus.
2. Demonstration: Instructors introduced dental equipment and demonstrated standardized clinical operations on dental simulators.
3. Practice: Students practiced on imitation head models under instructor supervision, receiving immediate technical guidance.

#### Experimental group (hybrid CBL-SST-simulator teaching)

Group A received a hybrid teaching mode integrating Case-Based Learning (CBL), Scenario Simulation Teaching (SST), and Dental Simulator practice. The teaching was divided into the following phases:

1. Pre-class rreparation (online): Instructors selected representative standardized patient medical records and uploaded case abstracts and thinking questions to an online learning platform. Students were required to complete this self-directed learning module approximately 3 days prior to each session to ensure adequate preparation.
2. Introduction and theory review (15 minutes): The instructor briefed students on the clinical scenario context. Key diagnostic logic and operational steps were reviewed, followed by an introduction to the setup of the dental simulator and instruments.
3. Simulated clinical encounter (CBL and SST) (30 minutes): Students worked in small groups to engage in role-playing exercises. Guided by CBL, the "Dentist" collected medical history and simulated visual/thermal tests on the "Patient." Then explained the diagnosis and treatment plan. Communication skills were practiced using the SEGUE framework.
4. Standardized operation on simulator (40 minutes): Students performed individual practices on dental simulator. Instructors circulated to provide guidance on posture and technique.
5. Feedback and evaluation (20 minutes): "Patients" provided feedback on the "Dentist’s" empathy and communication. Instructors evaluated the shape of the access cavity and pulp exposure on the models. The group discussed the differential diagnosis logic based on the CBL review.
6. Summary and consolidation (15 minutes): The instructor summarized common errors observed during diagnosis and operation. The session concluded with an assignment requiring students to write a complete outpatient medical record based on the simulated case.

### Evaluation system

Outcomes were assessed across four dimensions. All assessments were conducted one week after the conclusion of the course. Assessors were blinded to group allocation.

1. Theoretical knowledge: assessed via a written exam.
2. Clinical thinking: scored by the instructor based on the completeness of medical records, the logic of the consultation process, diagnosis/differential diagnosis, and the rationality of the treatment plan (Table 1).
3. Doctor-patient communication: assessed using the SEGUE Framework (Set the stage, Elicit information, Give information, Understand the patient, End the encounter), a validated instrument for this purpose [12].
4. Clinical skills: assessed via manual operations on dental simulators (Tooth 36 and 46) using a custom-designed rating scale based on the Chinese National Dental Licensing Examination standards.

**Table 1:**
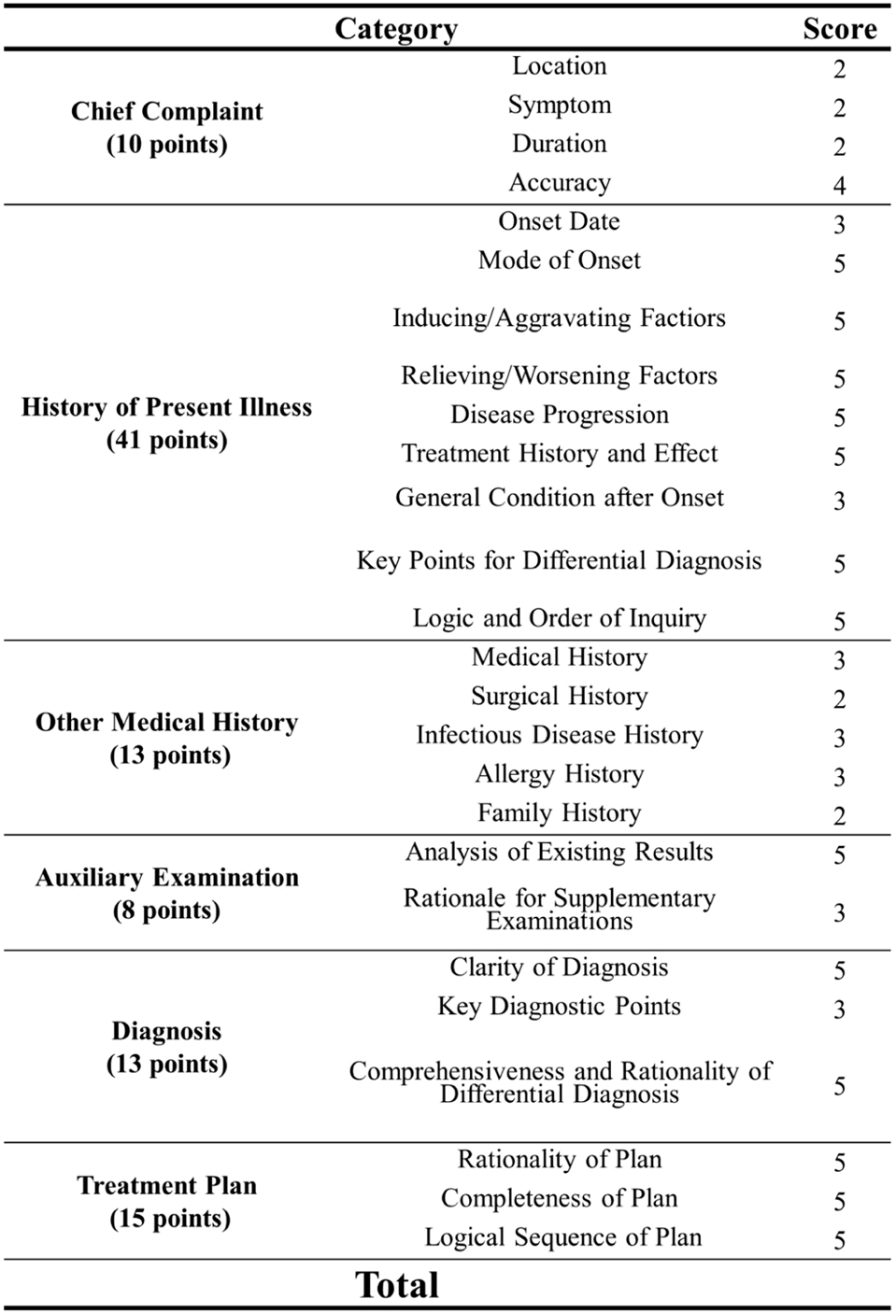
Scoring criteria for clinical thinking assessment

### Statistical analysis

Data are presented as mean ± standard deviation (SD). SPSS 25.0 software was used for analysis. The normality of the data distribution was assessed using the Shapiro-Wilk test. Continuous variables satisfying the assumption of normality were compared using the Independent Samples T-test, whereas non-normally distributed variables were analyzed using the Mann-Whitney U-test. A p-value of < 0.05 was considered statistically significant.

## Results

### Theoretical knowledge

**Fig. 1:**
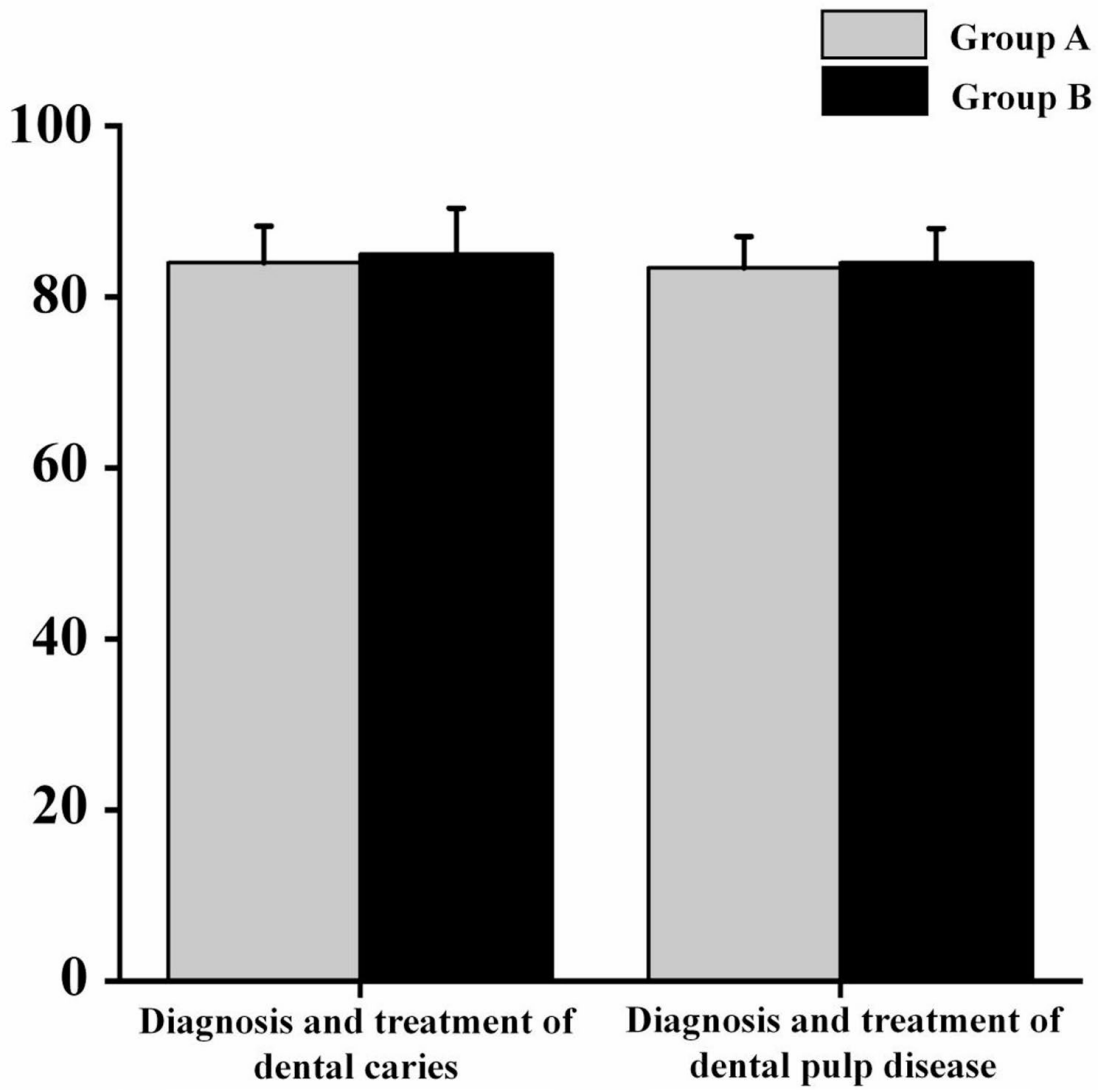
Analysis of theoretical exam scores between Group A (Hybrid) and Group B (LBL) (*P* > 0.05)

There was no significant difference in theoretical exam scores between Group A (Hybrid) and Group B (LBL) (*P* > 0.05). Both groups demonstrated adequate mastery of factual knowledge (Fig. 1).

### Clinical thinking

Group A scored significantly higher than Group B in the total clinical thinking assessment (87.40 ± 5.20 vs. 73.90 ± 8.41, *P* < 0.05). Sub-analysis showed that Group A performed better in “Diagnosis” and “Treatment Planning” domains, indicating a stronger ability to synthesize clinical information (Table 2).

**Table 2:**
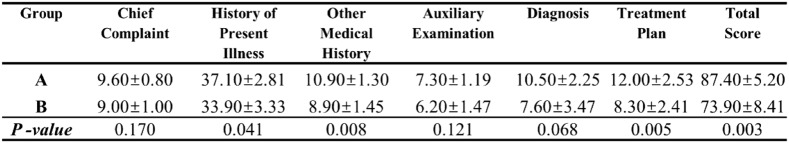
Analysis of the evaluation results of the clinical thinking assessment (score, mean ± SD)

Indicates a statistically significant difference in the total clinical thinking assessment scores between the Group A (Hybrid) and Group B (LBL) (P < 0.05)

### Doctor-patient communication

Using the SEGUE Framework, Group A significantly outperformed Group B (Total Score: 17.80 ± 1.89 vs. 14.20 ± 2.18, *P* < 0.05). Specifically, Group A showed superior performance in “Eliciting information” and “Understanding the patient’s perspective” (*P* < 0.05) (Table 3).

**Table 3:**
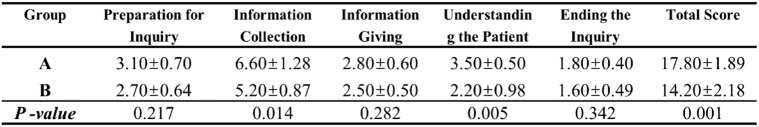
Analysis of the evaluation results of doctor-patient communication skills (score, mean ± SD)

Suggesting that there was statistically significant difference in the clinical thinking assessment between the Group A (Hybrid) and Group B (LBL) (P < 0.05)

### Clinical skills

**Fig. 2:**
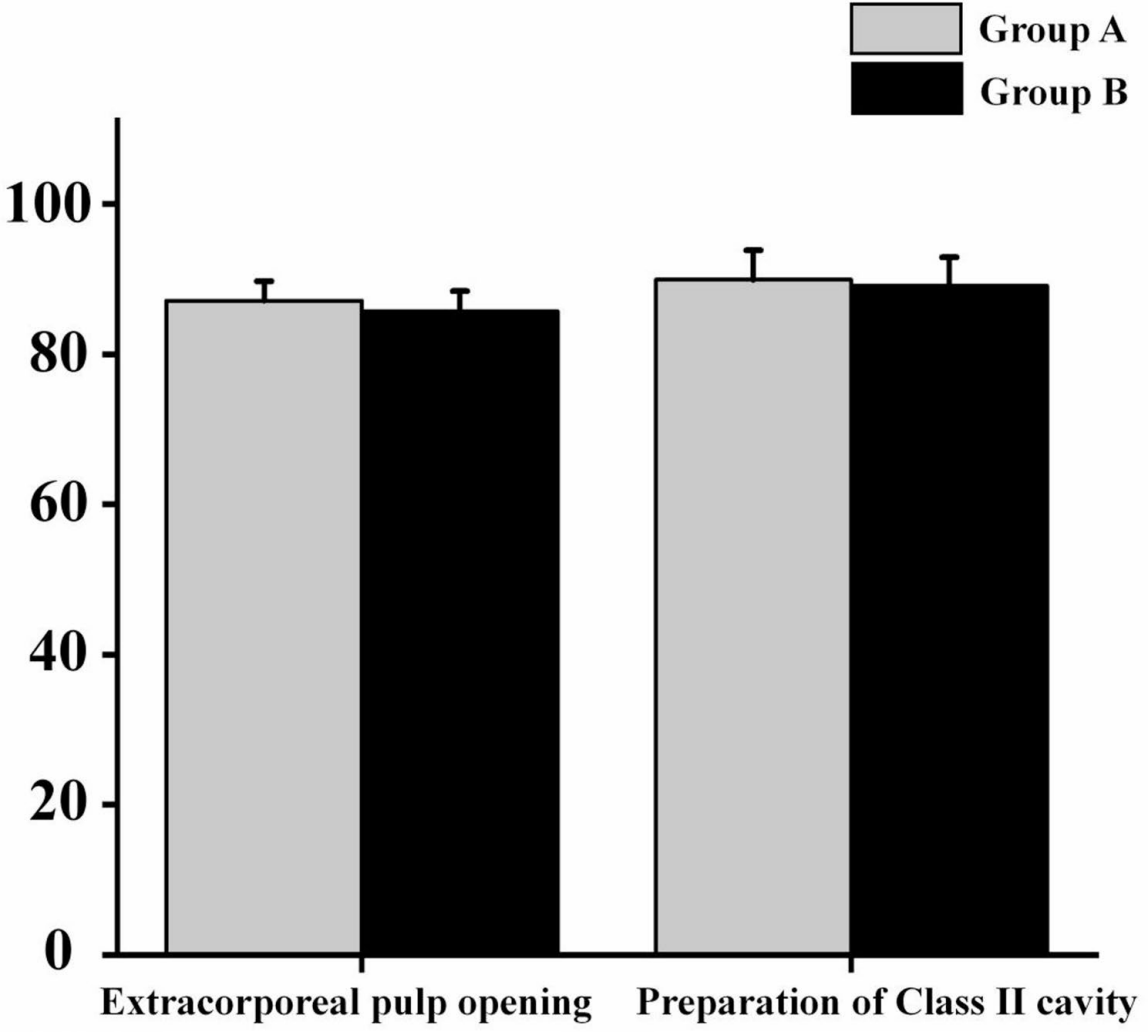
Results of clinical skills in the Group A (Hybrid) and Group B (LBL) (*P* > 0.05)

No significant difference was found between the groups in the technical quality of cavity preparation or access opening (*P* > 0.05). Both groups achieved passing standards for manual dexterity (Fig. 2).

## Discussion

This study explored the efficacy of a hybrid teaching model integrating CBL, SST, and dental simulator practice. Our findings reveal a nuanced impact: while the novel approach did not surpass traditional LBL in theoretical knowledge or manual dexterity, it demonstrated marked superiority in fostering clinical thinking and doctor-patient communication skills. These results align with recent calls for educational transformation in dentistry [13–15].

### The paradox of theoretical knowledge

The absence of significant differences in theoretical scores in our study presents an interesting contrast to recent aggregate data. A growing body of systematic reviews indicates that CBL typically yields superior theoretical scores compared to traditional lectures in dental education [3, 15, 16], However, our findings align with the meta-analysis by Shi et al. [14], which found no significant difference in theoretical scores between case-based flipped classrooms and LBL among students in Western contexts. This discrepancy may be attributed to several factors. First, Traditional LBL remains highly effective for delivering structured information, and conventional written exams primarily assess information recall rather than synthesis. Second, the theoretical exam was a part of the assessments required for course completion, so self-directed learning may have masked any potential differences in knowledge acquisition between the teaching models. According to Miller’s Pyramid of Clinical Competence [10], the theoretical exam primarily assesses the “Knows” and “Knows How” levels, which LBL covers adequately. However, the hybrid model targets the higher levels of “Shows How” and “Does,” which explains why differences appeared in clinical thinking rather than written exams.

### Enhancing higher-order cognitive skills

The substantial improvement in clinical thinking in the CBL-SST group can be interpreted by Cognitive Load Theory (CLT) [17]. CLT posits that working memory is limited and categorizes cognitive load into three types: intrinsic (inherent task complexity), extraneous (generated by ineffective instructional design), and germane (effort dedicated to schema construction) [18].

Endodontics, characterized by complex canal anatomy, imposes a naturally high intrinsic load. In the LBL group, students were required to mentally visualize 3D root canal structures from 2D slides while passively listening, likely generating high extraneous load that competed for working memory resources. Conversely, the Hybrid model optimized these loads. The pre-class case review allowed students to manage intrinsic load beforehand. During the session, the “Interactive” nature of SST and the physical realism of the simulator reduced extraneous load by aligning mental models with physical reality. This redistribution of cognitive resources favored germane load—the conscious processing required to link theory with practice—thereby facilitating the construction of sophisticated clinical schemas. This aligns with recent findings by Huang et al., who noted that blended approaches effectively balance cognitive demands [19].

### Cultivating empathy and professionalism

The significant improvement in SEGUE Framework scores (17.80 vs. 14.20) highlights the superiority of the SST component in addressing the humanistic aspects of dentistry. Effective communication is notoriously difficult to teach via didactic lectures alone, as noted in a recent literature review by Ho et al. [20], which advocates for experiential learning strategies. Our hybrid model provided a “psychologically safe” environment where students could experiment with phrasing and non-verbal cues without the risk of harming a real patient. This aligns with Moore’s narrative review, which suggests that communication skills are best acquired when technical training is integrated with patient interaction scenario [21]. By simulating the “Dentist-Patient” dynamic, students in the experimental group were forced to translate technical jargon into patient-friendly language, a skill that the LBL group had no opportunity to practice.

### Re-evaluating the assessment of clinical skills

The lack of difference in operational skills highlights the distinction between psychomotor skill acquisition and clinical translation. Manual dexterity relies heavily on “Deliberate Practice” and repetitive reinforcement [22]. Since both groups received equivalent time for simulator practice, their psychomotor skill acquisition was similar. The CBL-SST model fosters the ability to know *when* and *why* to apply a skill. While both groups learned to *perform* the skill (Miller’s ‘shows how’), the experimental group practiced applying it in a simulated context (‘does’) [10]. This suggests that assessment tools should evolve beyond simple checklists to process-oriented tools like the Objective Structured Assessment of Technical Skills (OSATS) [23].

### Limitations

Several limitations should be noted. First, although practical sessions were conducted separately, participants belonged to the same academic cohort. Informal information exchange outside of class could not be strictly controlled, posing a potential risk of contamination. Second, the small sample size (*N* = 20), which reflects the constraints of a pilot study within a single academic cohort. This study primarily evaluated Level 1 (Reaction) and Level 2 (Learning) of the Kirkpatrick Model [24]. We did not assess Level 3 (Behavior) or Level 4 (Results) in actual patient care, which warrants future longitudinal studies. While the hybrid model proved superior, it is resource-intensive, requiring simulators and standardized patients. Future research should explore cost-effective implementation strategies.

### Future Directions

While this study focused on Endodontics, the hybrid CBL-SST model holds significant potential for other dental disciplines. Complex fields such as Prosthodontics and Orthodontics, which require multifaceted treatment planning and extensive patient communication regarding costs and outcomes, would likely benefit even more from this integrated approach. Future studies should explore the application of this model in these broader contexts with larger sample sizes. Integrating technologies such as Virtual Reality (VR) and AI-powered virtual patients could further enhance realism [25, 26].

## Conclusions

This study provides evidence that a hybrid teaching model combining CBL, SST, and dental simulator practice is superior to traditional LBL for developing clinical thinking and doctor-patient communication skills. While not replacing LBL for foundational knowledge, this integrated, simulation-based approach represents a powerful strategy to prepare future dentists for the demands of modern clinical practice.

Tables.

Indicates a statistically significant difference in the total clinical thinking assessment scores between the Group A (Hybrid) and Group B (LBL) (*P* < 0.05).

Suggesting that there was statistically significant difference in the clinical thinking assessment between the Group A (Hybrid) and Group B (LBL) (*P* < 0.05).

## Supplementary Information

Supplementary Material 1.

Supplementary Material 2.

## Data Availability

The datasets used and/or analyzed during the current study are available from the corresponding author on reasonable request.
