# Supplementary material for: Efficacy of a hybrid case-based learning and simulated clinical encounter model versus lecture-based learning in dental education
Source: BMC Med Educ. 2026 Mar 9;26:620. doi: 10.1186/s12909-026-08937-x (PMC13085473; doi:10.1186/s12909-026-08937-x)
Supplement: Supplementary file 1 — Supplementary Material 1. [file 12909_2026_8937_MOESM1_ESM.docx]

**Answer Explanations for Oral Medicine (Endodontics) and (Cariology) Test Papers**

**Reference Answers for Oral Medicine (Endodontics) Test Paper**

# Single Choice Questions (2 points each, total 30 points)

1. C 2. D 3. C 4. B 5. A 6. A 7. B 8. B 9. B 10. B

11. C 12. B 13. B 14. C 15. D

# II. Multiple Choice Questions (3 points each, total 15 points)

1.ABCD 2. ABCE 3. ABCD 4. BC 5. ABCDE

# III. Noun Explanations (5 points each, total 20 points)

1. **Retrograde Pulpitis**: An inflammatory disease of the dental pulp caused by the retrograde invasion of infection into the pulp through a deep periodontal pocket. The affected tooth has a clear history of periodontitis and a deep periodontal pocket, with clinical manifestations similar to acute and chronic pulpitis.
2. **Physiological Apical Foramen**: Located at the apex of the tooth root, it is a natural passage between the dental pulp tissue and the periodontal tissue. It is the junction of dentin and cementum at the apex, and is a key structure for the blood supply and nerve entry and exit of the dental pulp.
3. **RCP (Root Canal Preparation Termination Point)**: Also known as the apical stop, corresponding to the apical constriction. It is the boundary for preparation and obturation in root canal treatment, which can prevent obturation materials from exceeding the apical foramen and damaging the periodontal tissue.
4. **Pulp Capping**: A treatment method to preserve vital pulp. A biocompatible pulp capping agent is used to cover the exposed or soon-to-be-exposed pulp surface to protect the pulp and promote the formation of reparative dentin. It is divided into direct pulp capping and indirect pulp capping.

# IV. Short Answer Questions (7 points each, total 35 points)

1. **Emergency Treatment Measures for Acute Pulpitis**

Pulp Chamber Opening and Drainage: Grind away the carious tissue to penetrate the pulp chamber, release the high pressure in the pulp chamber, and quickly relieve pain.

Sedation and Analgesia: Place sedative drugs such as clove oil in the pulp chamber to isolate external stimuli and relieve pain.

Occlusal Adjustment and Grinding: Appropriately reduce the occlusal height of the affected tooth to reduce pain caused by occlusal trauma.

Systemic Medication: When pain is severe, oral antibiotics and analgesics can be taken under the guidance of a doctor for auxiliary treatment.

1. **Differential Diagnosis Points Between Reversible Pulpitis and Irreversible Pulpitis**

Spontaneous Pain: Reversible pulpitis has no spontaneous pain; irreversible pulpitis has spontaneous pain, which is severe during acute attack.

Response to Pulp Temperature Test: Reversible pulpitis shows transient sensitivity to cold and heat stimulation, and symptoms disappear immediately after the stimulation is removed; irreversible pulpitis has prolonged pain after stimulation, and may also show the manifestation of pain relieved by cold and aggravated by heat.

Pulp Status and Treatment: The pulp of reversible pulpitis is in a reversible inflammatory state, and the treatment is mainly indirect pulp capping or sedation; the pulp inflammation of irreversible pulpitis is irreversible, requiring root canal treatment or vital pulpotomy for young permanent teeth.

1. **Operational Points of Pulp Temperature Test**

Before the test, isolate the saliva of the affected tooth and dry the tooth surface with cotton rolls to avoid saliva affecting the test results.

Follow the principle of "testing the control tooth first, then the affected tooth", and the control tooth is preferably the contralateral homologous tooth.

For cold test, ice sticks or ethyl chloride can be used; for heat test, hot gutta-percha is used, and the probe is placed at the middle 1/3 of the labial or buccal surface of the crown.

Observe and record the patient's response to judge the pulp vitality status.

1. **Indications and Principles of Vital Pulpotomy**

Indications: Young permanent teeth with incompletely developed apical foramen; pulp exposure with infection limited to the coronal pulp; young permanent teeth with short exposure time and small exposure hole due to trauma.

Principle: Resect the infected coronal pulp tissue, retain the healthy radicular pulp tissue, cover the root canal orifice with a pulp capping agent, promote the completion of apical foramen development, and retain the physiological function of the affected tooth.

1. **Causes of False Positive and False Negative Results in Pulp Electrical Vitality Test**

Causes of False Positive: The probe contacts the gingiva or metal restoration, forming an electrical circuit; the patient is overly nervous during the test, resulting in subjective illusion; the affected tooth has just been subjected to cold or heat stimulation, and the pulp is in a congested state; irritating drugs have been used on the affected tooth before the test.

Causes of False Negative: The pulp has necrosed and cannot respond to electrical stimulation; the apical foramen of young permanent teeth is not closed, the pulp blood supply is special, and it is not sensitive to electricity; the probe does not effectively contact the tooth surface, or the tooth surface is covered with a lot of tartar and plaque; the patient has taken sedatives or anesthetics before the test.

**Reference Answers for Oral Medicine (Cariology) Test Paper**

# Single Choice Questions (2 points each, total 30 points)

1. C 2. A 3. B 4. A 5. D 6. C 7. D 8. A 9. B

10. B 11. C 12. B 13. D 14. E 15. A

# II. Multiple Choice Questions (3 points each, total 15 points)

1.ABDE 2. ABCDE 3. ABDE 4. ABCDE 5. ABCDE

# III. Noun Explanations (5 points each, total 20 points)

1. **Dentino-Pulp Complex**: The dental pulp and dentin are closely connected in embryonic development, structure and function, and are collectively referred to as the dentino-pulp complex. The dentinal tubules contain odontoblastic processes, which are connected to the dental pulp. External stimuli can be transmitted to the pulp through the tubules to trigger reactions.
2. **Secondary Dentin**: After tooth eruption, under normal physiological conditions, the pulp tissue continuously forms dentin. It is an age-related change of dentin, which gradually thickens with age and reduces the volume of the pulp chamber.
3. **Remineralization Therapy**: A treatment method that uses artificial methods to remineralize demineralized hard tooth tissue and restore its hardness and structure. It is suitable for early enamel caries. By topical application of fluoride-containing preparations, etc., the further development of caries is prevented.
4. **Rampant Caries**: Also known as acute caries, it is an acute progressive type of caries. It is characterized by the simultaneous occurrence of caries on multiple teeth and multiple tooth surfaces in a short period of time, with a fast progression rate. It is common in children, adolescents, pregnant women or patients with systemic diseases with reduced saliva secretion.

# IV. Short Answer Questions (7 points each, total 35 points)

**1. Four-Factor Theory of Caries**

Bacteria: The main pathogenic bacteria for caries, mainly Streptococcus mutans, which can metabolize carbohydrates to produce acidic substances.

Food: Mainly carbohydrates such as sucrose, which provide energy for bacterial metabolism, and their metabolites can cause demineralization of hard tooth tissue.

Host: Including the shape, arrangement and mineralization degree of teeth, as well as the flow rate and composition of saliva. These factors affect the occurrence and development of caries.

Time: The occurrence of caries requires a certain period of time. Bacteria need to continuously act on the tooth surface to produce acid, and caries cavities will form after long-term demineralization.

1. **Differential Diagnosis and Treatment Principles of Deep Caries**

Differential Diagnosis: Differentiate from reversible pulpitis, which has transient spontaneous pain, sensitive to temperature test with short duration; differentiate from irreversible pulpitis, which has spontaneous pain, persistent and severe pain in temperature test; differentiate from pulp necrosis, which has no response to cold and heat test, and the tooth may discolor.

Treatment Principles: Remove all carious tissue to eliminate the source of infection; strictly protect the pulp during operation to avoid mechanical and chemical stimulation; select treatment plan according to pulp status. For patients with normal but sensitive pulp vitality, indirect pulp capping or sedation treatment is performed; for patients with infected and necrotic pulp, root canal treatment is required.

1. **Basic Principles of Cavity Preparation**

Remove All Carious Tissue: Completely remove carious tissue, eliminate infection, and prevent recurrence of caries.

Protect the Pulp: Avoid excessive cutting and chemical stimulation during operation, and use pulp protective materials if necessary.

Retain Healthy Tooth Tissue: Minimize the grinding of healthy tooth tissue to maintain the physiological strength of the tooth.

Prepare Retention Form and Resistance Form: Ensure that the filling can be stably retained, and at the same time enhance the fracture resistance of the tooth to avoid tooth fracture after filling.

1. **Adhesion Mechanism of Composite Resin Restoration**

Etching Stage: The etchant demineralizes the enamel surface to form a microscopic pore structure, increasing the adhesion area of the tooth surface.

Penetration Stage: The adhesive penetrates into the micropores formed after etching.

Curing Stage: After curing, the adhesive forms a micromechanical interlock with the tooth tissue, and at the same time, the adhesive forms a chemical bond with the composite resin matrix, finally realizing the firm adhesion between the composite resin and the tooth tissue.

1. **Arrested Caries and Its Occurrence Conditions**

Arrested Caries: A type of caries in which the development of the lesion stops and remains unchanged for a long time due to the disappearance of cariogenic factors caused by environmental changes after the caries develops to a certain stage.

Occurrence Conditions: The carious lesion is exposed, and saliva can directly wash and clean it; the eruption position of the tooth changes, eliminating the condition of food impaction; the oral hygiene status is significantly improved, and the number of cariogenic bacteria is greatly reduced.
