# Supplementary material for: Efficacy of a hybrid case-based learning and simulated clinical encounter model versus lecture-based learning in dental education
Source: BMC Med Educ. 2026 Mar 9;26:620. doi: 10.1186/s12909-026-08937-x (PMC13085473; doi:10.1186/s12909-026-08937-x)
Supplement: Supplementary file 2 — Supplementary Material 2. [file 12909_2026_8937_MOESM2_ESM.docx]

**Oral Medicine (Endodontics) Test Paper**

**Exam Duration: 60 minutes**

**Total Score: 100 points**

# I. Single-Choice Questions (2 points each, 30 points total)

Please select the best answer from the alternatives for each question.

1. What is the most important pathogenic factor of pulpitis?
2. Physical factors (trauma, temperature)
3. Chemical factors (irritation from filling materials)
4. Bacterial factors (mainly anaerobic bacteria)
5. Immune factors
6. Idiopathic factors
7. Which of the following is NOT a typical pain characteristic of acute pulpitis?
8. Spontaneous, paroxysmal pain
9. Night pain
10. Pain exacerbated by temperature stimulation
11. Precise localization of pain
12. Radiating pain
13. What is the most reliable clinical examination method for diagnosing pulpitis?
14. Visual inspection
15. Percussion
16. Pulp vitality temperature test (cold-hot test)
17. Probing
18. X-ray examination
19. What is a characteristic of chronic ulcerative pulpitis?
20. Severe spontaneous pain
21. Exposed pulp hole detected in the cavity with obvious probing pain
22. Diminished response to temperature testing
23. Precise pain localization
24. No food impaction pain
25. What is the source of infection in retrograde pulpitis?
26. Deep periodontal pocket
27. Deep carious cavity
28. Dental crown crack
29. Malformed central cusp
30. Hematogenous infection
31. What is the preferred emergency treatment for acute pulpitis?
32. Pulp chamber opening and drainage
33. Extraction of the affected tooth
34. Administration of antibiotics
35. Local block injection
36. Occlusal adjustment and grinding
37. In pulp temperature testing, what is the usual response of normal pulp to cold stimulation?
38. No response
39. Transient mild pain or discomfort that disappears after stimulation is removed
40. Severe persistent pain
41. Ipsilateral headache
42. None of the above
43. For young permanent teeth (with incompletely developed apical foramen) with pulpitis, what is the preferred treatment method?
44. Root canal therapy
45. Pulpotomy
46. Mummification therapy
47. Resinifying therapy
48. Empty canal therapy
49. What is the most important clinical examination for diagnosing pulp necrosis?
50. Visual inspection of tooth color changes
51. Cold-hot test
52. X-ray film
53. Pulp electric vitality test
54. Percussion
55. Which of the following is NOT a characteristic of chronic pulpitis?
56. Usually clear localization of the affected tooth
57. Severe night pain
58. History of radiating pain
59. Spontaneous dull or vague pain
60. Diminished response to cold-hot testing
61. What is the most valuable diagnostic indicator for residual pulpitis?
62. Apical radiolucency on X-ray film
63. No response to temperature testing
64. Pain or sensation when probing the deep root canal after removing the original filling material
65. Percussion pain
66. Tooth mobility
67. Resinifying therapy is mainly indicated for?
68. Young permanent teeth with unclosed apical foramen
69. Permanent molars with pulp necrosis and closed apical foramen
70. Anterior teeth
71. Primary teeth
72. Traumatized teeth with exposed pulp
73. What is a commonly used pulp devitalizing agent?
74. Eugenol
75. Paraformaldehyde
76. Phenolic resin
77. Camphorated phenol
78. Sodium hypochlorite
79. What is the main differentiating point between the serous stage of acute apical periodontitis and acute pulpitis?
80. Nature of pain
81. Pulp vitality (whether necrotic)
82. Occlusal pain (a floating sensation and occlusal pain in the affected tooth)
83. Response to temperature stimulation
84. Ability to localize pain
85. What is the most commonly used method for diagnosing pulp diseases?
86. X-ray film
87. CBCT
88. Electric vitality test
89. Temperature test
90. Probing and percussion

# Multiple-Choice Questions (3 points each, 15 points total)

Each question has 2 or more correct answers; select at least 2 answers.

1. Physical factors causing pulpitis include:
2. Trauma (occlusal trauma)
3. Temperature (heat generated during cavity preparation)
4. Electric current (dissimilar metal restorations)
5. Laser
6. Drug corrosion
7. The pain nature of acute pulpitis can manifest as:
8. Sharp pain
9. Throbbing pain (pulsatile pain)
10. Radiating pain
11. Dull pain
12. Burning pain
13. Indications for root canal therapy include:
14. Irreversible pulpitis
15. Pulp necrosis
16. Apical periodontitis
17. Periodontal-endodontic lesions
18. Cracked teeth requiring full crown restoration for protection
19. Methods for determining pulp vitality include:
20. Visual inspection (observing color)
21. Pulp temperature test
22. Pulp electric vitality test
23. Percussion
24. X-ray examination
25. Pain characteristics of pulp diseases include ():
26. Acute pulpitis often presents with spontaneous, paroxysmal severe pain
27. Chronic pulpitis generally has a long history of sensitivity to cold and hot stimulation
28. Acute suppurative pulpitis has exacerbated pain with hot stimulation and relieved pain with cold stimulation
29. Pulp calcification may present with spontaneous pain related to body position
30. The pain characteristics of residual pulpitis are similar to those of general chronic pulpitis but with a history of pulp treatment

# III. Term Explanations (5 points each, 20 points total)

1. Retrograde pulpitis
2. Physiological apical foramen
3. RCP (Retruded Contact Position)
4. Pulp capping

# IV. Short Answer Questions (7 points each, 35 points total)

1. Briefly describe the emergency treatment measures for acute pulpitis.
2. Briefly describe the differential diagnosis points between reversible pulpitis and irreversible pulpitis.
3. Briefly describe the operational points of the pulp temperature test.
4. Briefly describe the indications and principles of pulpotomy.
5. What are the causes of false positive and false negative results in the pulp electric vitality test?

**Oral Medicine (Cariology) Test Paper**

**Exam Duration: 60 minutes**

**Total Score: 100 points**

# Single-Choice Questions (2 points each, 30 points total)

Please select the best answer from the alternatives for each question.

1. Which type of caries is classified by the degree of damage?
2. Acute caries
3. Chronic caries
4. Moderate caries
5. Arrested caries
6. Fissure caries
7. What is the most important pathogenic bacterium in the development of caries?
8. Streptococcus mutans
9. Lactobacillus
10. Actinomyces
11. Veillonella
12. Peptostreptococcus
13. In the permanent dentition, which tooth usually has the highest caries prevalence and is the earliest affected?
14. Maxillary first molar
15. Mandibular first molar
16. Mandibular second molar
17. Maxillary anterior teeth
18. Mandibular anterior teeth
19. Which is the most common site for early caries?
20. Occlusal fissures
21. Below the proximal contact point
22. Cervical margin
23. Lingual surface
24. Root surface
25. What is the most accurate method for diagnosing early proximal caries?
26. Visual inspection
27. Probing
28. Occlusal test
29. X-ray film (e.g., bitewing film)
30. Temperature test
31. Which of the following is NOT a clinical manifestation of moderate caries?
32. Cavity formation
33. Sensitivity to cold, hot, sour, and sweet stimuli
34. Spontaneous pain
35. Symptoms disappear immediately after stimulation is removed
36. Caries lesion reaches the superficial dentin
37. What is the mechanism of acid-etch bonding?
38. Chelate bonds
39. Chemical bonds
40. Van der Waals forces
41. Micromechanical interlocking
42. Copolymerization
43. When filling with composite resin, where should the cavity margin bevel be prepared?
44. Enamel at the cavity margin
45. Dentin at the cavity margin
46. Cementum at the cavity margin
47. Dentin at the gingival margin
48. Any location
49. For young permanent teeth with localized or reversible pulp lesions, the preferred treatment method is?

A. Root canal therapy

B. Indirect pulp capping

C. Direct pulp capping

D. Pulpotomy

E. Mummification of dental pulp

1. GV Black Class IV cavity refers to ():
2. Cavities prepared for caries lesions in the cervical 1/3 of the labial or buccal surfaces of all teeth
3. Cavities prepared for caries lesions on the proximal surfaces of anterior teeth involving the incisal angle
4. Cavities prepared for caries lesions on the proximal surfaces of posterior teeth
5. Cavities prepared for caries lesions on the proximal surfaces of anterior teeth not involving the incisal angle
6. Cavities prepared for caries lesions on the occlusal surfaces of teeth
7. Which material has the ability to release fluoride ions, helping to prevent secondary caries?
8. Amalgam
9. Composite resin
10. Glass ionomer cement
11. Zinc phosphate cement
12. Polycarboxylate zinc cement
13. During the treatment of deep caries, if the patient's pulp vitality is normal but sensitive, what is the preferred treatment plan?
14. Direct pulp capping
15. Indirect pulp capping/comforting treatment
16. Root canal therapy
17. Extraction
18. Drug therapy
19. Which of the following is NOT a clinical characteristic of acute caries?
20. Light color of the lesion tissue
21. Soft and moist texture
22. Rapid progression
23. Abundant formation of reparative dentin
24. Easy excavation
25. Indications for preventive resin restoration do NOT include?
26. Fissures with caries that can trap a probe
27. Deep fissures with caries susceptibility
28. Early signs of caries in fissures
29. Caries in the contralateral homologous tooth
30. Caries cavities that have deepened to the pulp
31. Which of the following practices does NOT help improve the resistance form of fillings?
32. Preparing undercuts to form a structure that is wider at the bottom and narrower at the top
33. Grinding away unsupported enamel
34. Preparing a box-shaped cavity
35. Preparing a depth of 0.2-0.5mm below the enamel-dentin junction
36. Removing thin and weak cusps at the cavity margin

# Multiple-Choice Questions (3 points each, 15 points total)

Each question has 2 or more correct answers; select at least 2 answers.

1. Clinical characteristics of caries include:
2. Color, shape, and texture changes of dental hard tissues
3. Progressive development
4. Spontaneous pain and night pain in the affected tooth
5. Predilection for sites such as fissures and proximal surfaces
6. Often no subjective symptoms in the early stage
7. Factors affecting the occurrence and development of caries include:
8. Salivary flow rate and composition
9. Tooth arrangement and mineralization degree
10. Dietary habits (e.g., sugar intake)
11. Oral hygiene status
12. Genetic factors
13. Advantages of composite resin filling include:
14. Aesthetics, color similar to natural teeth
15. Good adhesion to dental hard tissues
16. Excellent physical and mechanical properties, wear resistance exceeding amalgam
17. Can be used for minimally invasive restoration of various dental defects
18. Minimal cutting of dental hard tissues
19. Cavity forms that can effectively improve the retention of fillings include:
20. Step retention form
21. Sidewall retention form
22. Dovetail retention form
23. Undercut retention form
24. Trapezoidal retention form
25. Which of the following measures help prevent caries?
26. Correct brushing and flossing
27. Using fluoride-containing toothpaste
28. Regular oral examinations
29. Fissure sealing
30. Controlling the frequency of sugar intake

# III. Term Explanations (5 points each, 20 points total)

1. Pulp-dentin complex
2. Secondary dentin
3. Remineralization therapy
4. Rampant caries

# IV. Short Answer Questions (7 points each, 35 points total)

1. Briefly describe the four-factor theory of caries.
2. Briefly describe the differential diagnosis and treatment principles of deep caries.
3. What are the basic principles of cavity preparation?
4. Briefly describe the bonding mechanism of composite resin restoration.
5. What is arrested caries? What are the conditions for its occurrence?
